# Synthesis of a new amino acid derivative with long‐lasting hair shape control effects and elucidation of its mechanisms

**DOI:** 10.1111/ics.13054

**Published:** 2025-02-18

**Authors:** Sotaro Sato, Hinako Tabuchi, Kohtaro Katayama, Kenji Matsumoto, Hiroyuki Miyamoto, Sorai Kanno, Hiroshi Sekiguchi, Yoshinobu Tanaka, Mari Inoue, Choji Murata, Hiroki Hotta, Yoshio Tsujino

**Affiliations:** ^1^ Graduate School of Maritime Sciences Kobe University Kobe Japan; ^2^ Graduate School of Science, Technology and Innovation Kobe University Kobe Japan; ^3^ Japan Synchrotron Radiation Research Institute (JASRI) Sayo‐gun Japan; ^4^ B. Products Taco Kobe Japan; ^5^ Graduate School of Human Development and Environment Kobe University Kobe Japan

**Keywords:** 2‐(2‐aminoethylthio) succinic acid, chemical analysis, chemical synthesis, hair straightening, hair styling effect, spectroscopy

## Abstract

**Objective:**

Hair is a crucial aspect of an individual's physical appearance. Thus, there is always a need for technology to care for curls and undulations. In this study, we found that heat treatment with a mixed aqueous solution of cysteamine (2‐aminoethanethiol) and fumaric acid was highly effective in improving hair quality. The purpose of this study was to scientifically elucidate the effectiveness of this technology by identifying its active ingredients, evaluating their functionality and analysing changes in the internal structure of hair after treatment.

**Method:**

We observed changes in hair shape through a hair straightening test, analysed the components using liquid chromatography/mass spectrometry (LC/MS) and investigated the effects on mechanical properties using bending and surface friction measurements. Furthermore, we analysed the hair conditions using small‐angle X‐ray scattering (SAXS) measurements and evaluated water retention using thermal analysis.

**Results:**

In the hair straightening test, even extremely strong curls of African human hair improved. The LC/MS results revealed that the active ingredient was 2‐(2‐aminoethylthio) succinic acid (ATS). Furthermore, by treating hair with ATS, decreases in the coefficient of friction and bending stiffness and an increase in moisture content were observed. The SAXS measurements revealed that the treatment widened the distance between the intermediate filaments (IFs) inside the hair and improved the orientation of the IFs.

**Conclusion:**

ATS, produced by the thiol–ene reaction between fumaric acid and cysteamine, acts as an active ingredient in hair shape control. ATS reacted within the matrix and increased the IF–IF distance. This suggests that ATS functions as a crosslinker for keratin proteins. ATS is believed to increase the moisture content of hair and improve hair texture.

## Graphical abstract

We have discovered 2‐(2‐aminoethylthio) succinic acid with a long‐lasting hair shape control effect. We have demonstrated its hair straightening and curl retention effect, and clarified its mechanism.

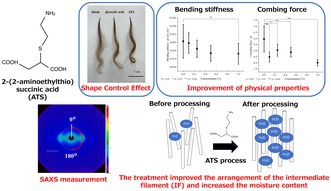

## Résumé

**Objectif:**

les cheveux sont un aspect crucial de l'apparence physique d'un individu. Il existe donc un besoin constant de technologies permettant de prendre soin des boucles et ondulations. Dans cette étude, nous avons découvert qu'un traitement thermique à l'aide d'une solution aqueuse mixte de cystéamine (2‐aminoéthanethiol) et d'acide fumarique était particulièrement efficace pour améliorer la qualité des cheveux. L'objectif de cette étude était d'élucider scientifiquement l'efficacité de cette technologie en identifiant ses ingrédients actifs, en évaluant leur fonctionnalité et en analysant les modifications de la structure interne des cheveux après le traitement.

**Méthode:**

Nous avons observé les changements de forme des cheveux à l'aide d'un test de lissage, analysé les composants à l'aide de chromatographie liquide‐spectrométrie de masse (LC/MS) et étudié les effets sur les propriétés mécaniques grâce aux mesures de flexion et de friction de surface. De plus, nous avons analysé l'état des cheveux à l'aide de la diffusion des rayons X aux petits angles (SAXS) et évalué la rétention d'eau grâce à l'analyse thermique.

**Résultats:**

Lors du test de lissage, même les boucles extrêmement serrées des cheveux humains africains ont été améliorées. Les résultats de la LC/MS ont révélé que l'ingrédient actif était l'acide 2‐(2‐aminoéthylthio) succinique (ATS). De plus, après un traitement des cheveux avec l'ATS, nous avons observé une diminution du coefficient de friction et de la rigidité en flexion ainsi qu'une augmentation de la teneur en eau. Les mesures SAXS ont révélé que le traitement élargissait l'espace entre les filaments intermédiaires (IFs) à l'intérieur des cheveux et améliorait leur orientation.

**Conclusion:**

l'ATS, produit par la réaction thiol‐ène entre l'acide fumarique et la cystéamine, agit comme un ingrédient actif dans le contrôle de la forme des cheveux. L'ATS réagit au sein de la matrice capillaire et augmente la distance entre les IFs, suggérant qu'il fonctionne comme un agent réticulant pour les protéines de kératine. L'ATS est supposé augmenter la teneur en eau des cheveux et améliorer leur texture.

## INTRODUCTION

Hair not only plays a role in life, such as a protective function against external stimuli like ultraviolet rays and friction, but also plays a key role in one's physical appearance. In recent years, both women and men have become more interested in beautiful hair, and there is a constant demand for techniques that can treat not only the damage caused by chemical treatments such as perms, hair colouring and bleaching but also the curls and undulations that occur with ageing. A typical hair straightening technique uses a reducing agent solution adjusted for basicity to break the disulfide bonds in hair, heat treatment using iron and oxidation using an oxidizing agent [1]. However, this approach weakens and damages hair owing to the effects of alkalis, heat during treatment and mixed disulfides produced during the reduction step [2]. To mitigate this damage, a method was devised to reduce the damage by mixing two component‐reducing agents [3, 4]. Alkaline agents used for strong hair straightening cause significant damage to hair because of the generation of irreversible lanthionine crosslinks, whereas two‐component systems that replace alkaline agents cause less damage because the reduction reaction with thioglycolic acid is reversible. However, hair damage is still unavoidable, and new treatment methods must be developed. The use of formaldehyde to form a crosslinked structure within the hair and generate a curling effect has also been reported [5]. Although this method is inexpensive and can quickly straighten hair, it is subject to strict regulations due to problems such as cancer, respiratory system abnormalities and allergies caused by exposure [6]. Therefore, there is a strong demand for safe and environment‐friendly alternative substances. Recently, bioconjugation technology has attracted attention for creating new crosslinked structures within hair and improving hair quality. Boga et al. used Raman scattering and Fourier transform‐infrared (FT‐IR) spectroscopy to measure changes in the secondary structure and disulfide crosslinking of hair treated with glyoxylic acid [7]. Leite et al. reported that the breaking strength of hair treated with formaldehyde was reduced by 30%, whereas treatment with glyoxylic acid resulted in a decrease of approximately 10% [8]. However, hair treated with glyoxylic acid has a unique odour and continued use will inevitably cause damage such as fading of hair colour and loss of protein [9, 10]. In addition, acute kidney damage due to the use of hair straighteners containing glyoxylic acid has been reported, leading some to call for their discontinuation for health reasons [11]. Although various hair straightening techniques have been developed, several issues remain to be addressed, such as damage to the hair, returning the hair to its original curled state after exposure to high humidity, deposition of odours due to processing and health hazards. On the other hand, we have recently found that a treatment solution prepared by heating a mixed aqueous solution of cysteamine and fumaric acid has a high shape‐memory effect on hair. In this study, we analysed this mixed solution to identify the active ingredients involved in hair straightening and attempted to elucidate their effects on hair using bending measurements, surface friction measurements, thermal analysis and small‐angle X‐ray scattering (SAXS) measurements.

## MATERIALS AND METHODS

### Materials

Deionized MilliQ water (>18.2 MΩ cm^−1^) was used for all experiments. Fumaric acid, hydrogen peroxide, acetic acid and sodium dodecyl sulfate were purchased from Fujifilm Wako Pure Chemical Corp., Osaka, Japan. 2‐aminoethanethiol (cysteamine) hydrochloride and ammonium thioglycolate were obtained from Tokyo Chemical Industry Co., Ltd., Tokyo, Japan. All the chemical reagents were used as received.

### Synthesis and analysis of novel functional components

First, 3.85 g of cysteamine and 5.80 g of fumaric acid were dissolved in 50 mL of water and heated under reflux. The products were analysed using a high‐performance liquid chromatography (HPLC) system equipped with a photodiode array detector (Alliance HPLC System; Waters, MA, USA). A 10 mM aqueous acetic acid solution was used as the eluent. ODS column (Inert Sustain C18, 5 μm, 4.6 × 150 mm, GL Sciences, Tokyo, Japan) was used as a separation column. The chemical structure of the product was determined using a quadrupole ion trap mass spectrometer (SCIEX, 4000 QTRAP LC‐MS/MS, MA, USA) and an AVANCE 500 digital NMR device (Bruker, MA, USA).

### Hair samples

Hair samples used were commercially available bleached hair (STAFFS Co., Ltd. Aichi, Japan) and African human hair (purchased from Agent Totsuka), which were washed with a 5% sodium dodecyl sulfate aqueous solution and dried using a hair dryer. Hair straightening treatment was performed using the following steps: As the first agent, a 10% thioglycolic acid aqueous solution (pH 9.0) was used. It was prepared by mixing a thioglycolic acid cold two‐bath perm agent (Salawara Cold Wave Lotion, Napla Co., Ltd. Osaka, Japan), a 50% ammonium thioglycolate aqueous solution, and purified water at a volume ratio of 1:1:3. At first, a hair bundle was immersed in the solution for 15 min, washed with water, lightly drained and then immersed in an 0%–1.0% of ATS aqueous solution (the second agent) for 5 min. After gently rinsing the hair with water again and heating it to the specified temperature with a hair iron, a third agent (2 vol% hydrogen peroxide aqueous solution, pH 6.1) was added for 7 min. The hair bundles were washed again and dried with cold air from a hair dryer.

### Microscopic observation and curl retention test

The surface conditions of the hair bundles were observed using a digital microscope (VHX‐8000, Keyence, Japan). African hair was placed on a glass slide, and both ends were secured with an adhesive tape. By marking the observed areas, we observed the changes in the same areas before and after the ATS treatment. In addition, a curl retention test was performed. A total of 0.4 g of hair (*L* = 25 cm) was immersed in each solution for 5 min, wrapped around a rod, and left for 15 min. The samples were then dried in a hair dryer for 15 min, removed from the rod and the length of the hair (*L*_0_) was measured. After leaving the hair in the constant temperature and humidity bath (KCL‐2000, Eyela, Japan) at 40°C and relative humidity (RH) 88% for 1 h, the hair length (*L*_t_) was measured again. Curl retention effect was calculated as (*L* − *L*_t_)/(*L* − *L*_0_) × 100.

### Friction measurement and combing test

Friction measurements were performed using a TL201Tt friction meter (Trinity Lab, Tokyo, Japan). Ten treated hair samples were arranged on a 26 mm × 70 mm glass slide at 1 mm intervals. In a glove box adjusted to a temperature of 25 ± 5°C and an RH of 50% ± 10%, three samples were measured with a load of 20 g and a speed of 1.4 mm s^−1^, and the average value of the coefficient of friction was determined. The contactor used for measurement was made of rubber, weighted and speed, according to the report of Bharat Bhushan et al. [12]. A comb test was also performed using the TL201Tt. Both ends of the hair bundle were fixed, and the comb was repeatedly passed 10 times in the direction from the root to the tip of the hair. The comb movement distance was 30 mm and the speed was 1.0 mm s^−1^.

### Bending stiffness measurement

Twenty hair samples were laid in parallel at 1 mm intervals and fixed at both ends with a piece of cardboard so that the measurement site was 1 cm in length and was used as the measurement sample. Measurements were performed using a KES‐FB2 type bending tester (maximum curvature ±2.5 cm^−1^, deformation speed 0.5 cm^−1^ s^−1^, Kato Tech, Kyoto, Japan) at a temperature of 25 ± 5°C and an RH of 50% ± 10%. A typical example of the change in the bending moment *M* with the bending curvature *K* is shown in Figure S1. In this experiment, bending stiffness with a slope of 0.5–1.5 cm^−1^ was calculated. To ensure the reliability of the measurement results, the measurements were repeated for 12 samples (*n* = 12), and the average value was used for comparison.

### Moisture content measurement

The hair moisture content was measured using a thermogravimetry/differential thermal analysis (TG/DTA) measuring device (DTG‐60, Shimadzu Co., Kyoto, Japan). The hair was cut into small pieces, and 5.0 mg of the sample was set into a measuring pan. The hair was kept for 1 day at a temperature of 25°C and an RH of 55% using a constant temperature and humidity bath. The measuring pans were immediately capped to keep them sealed, and a small hole was pierced just before measurement. The measurements were performed according to the method of Barba et al. [13], and the temperature was raised from 30°C to 65°C at a rate of 20°C min^−1^, held for 40 min, then raised again 20°C min^−1^ to 180°C.

### Small‐angle X‐ray scattering (SAXS) measurement

SAXS measurements were performed at BL40XU the large synchrotron radiation facility SPring‐8 (Hyogo, Japan). A high‐flux beam from a helical undulator with a wavelength of 0.083 nm was focused horizontally and vertically using two mirrors. A micro‐beam X‐ray with about 5 μm in diameter with a flux of 10^11^ photons/s was obtained by a 5 μm diameter pinhole installed 150 mm upstream from the sample. To fix the hair sample, a homemade holder with a 4 mm × 34 mm rectangular window in the centre of a 40 mm2, 2 mm thick acrylic plate was used. The hair was attached to the holder with an adhesive tape and fixed perpendicularly to the beam for the SAXS measurements. The SAXS measurements were performed with a sample‐detection distance of approximately 1500 mm, which was calibrated by using diffraction from silver behenate (layer spacing of 58.38 Å) and an exposure time of 1.0 s. An X‐ray image intensifier (V7739P, Hamamatsu Photonics) and CMOS camera (C11440‐22CU, Hamamatsu Photonics) were used as detectors. For measurements, the hair sample was moved diametrically in steps of 5 μm, and SAXS images were recorded at each position. The 1D scattering profiles for each azimuth angle were obtained by processing the 2D SAXS scattering images using PyFAI software [14]. The intermediate filament (IF) radius and IF–IF distance were determined from the SAXS images according to the method described by Briki et al. [15]. We also investigated the slope of IF from the scattering intensity in the azimuthal direction [16, 17].

## RESULTS AND DISCUSSION

### Heat treatment with the mixed solution of cysteamine and fumaric acid

**FIGURE 1:**
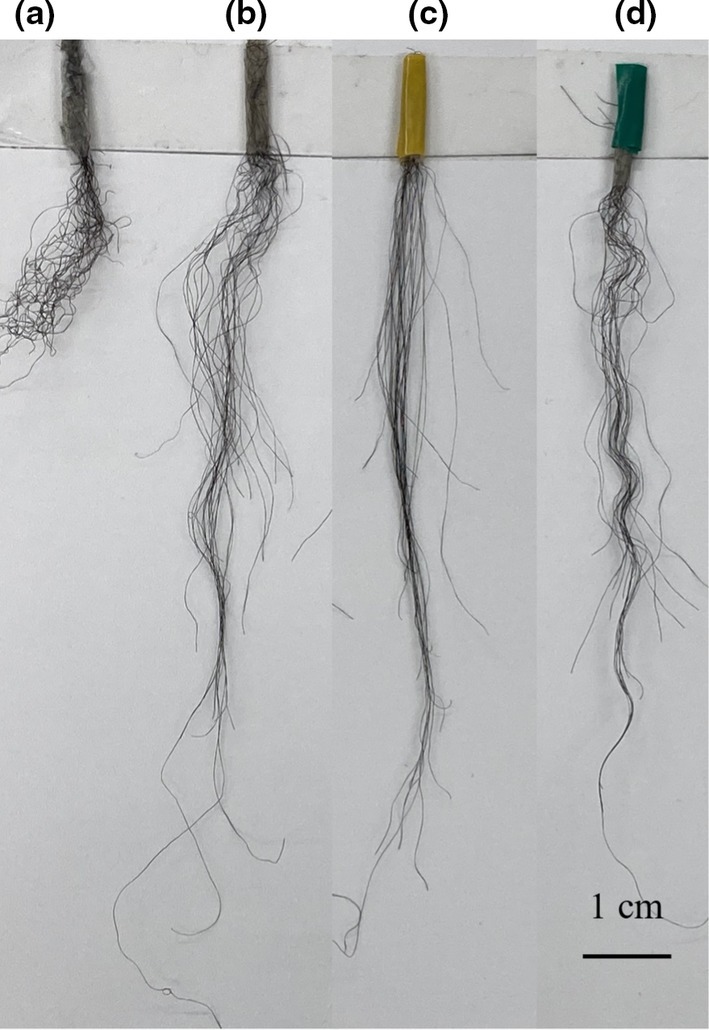
Changes in hair appearance due to each treatment. (a) Before treatment, (b) treatment with only water (without ATS), (c) treatment with a mixed solution of cysteamine and fumaric acid reaction products, (d) treated by succinic acid.

Experiments were conducted using strongly curled African human hair to verify the efficacy of the proposed treatment. Figure 1 shows the results. The sample before treatment was curly and appeared short (Figure 1a), but when it was immersed in a mixed aqueous solution of cysteamine and fumaric acid and treated with a hair iron at 120°C, the hair straightened out (Figure 1c). It became straighter than when it was immersed in pure water and treated with a hair iron at 120°C (blank treatment; Figure 1b). This hair‐straightening effect was not achieved by treatment with cysteamine alone, succinic acid alone or cysteamine and fumaric acid without heating by a hair iron. Therefore, the internal chemical structure of hair changed because of the reaction product produced by heating a mixed solution of cysteamine and fumaric acid. Moreover, this treatment was more effective at straightening hair than the acid‐heat treatment using succinic acid as shown in Figure 1d. This effect continued over time. This substance has been shown to have unprecedented and extremely strong effects on hair quality. The functional components of the treated solution were analysed.

### Detection of functional compounds

**FIGURE 2:**
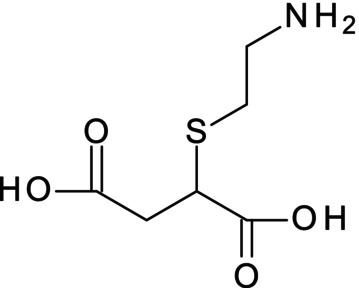
The structure of 2‐(2‐aminoethylthio) succinic acid, ATS.

The reaction products of a mixture of cysteamine and fumaric acid in water‐heated reflux for 1 h were analysed using LC/MS. The chromatogram of the reaction solution is shown in Figure S2. In addition to the raw material residue, the formation of a compound with a retention time of 2.2 min was confirmed. The positive‐ion mode mass spectrum of the compound is shown in Figure S3, where the reaction product was found to be a substance with *m/z* 192.16. The attributes of the fragment ions are shown in the figure. This fraction isolated was analysed by nuclear magnetic resonance (NMR) and electron ionization (EI) mass spectrometry measurements and was confirmed to be 2‐(2‐aminoethylthio) succinic acid [Figure 2; ^1^H NMR (500 MHz, D_2_O): *δ* = 2.52 (dd, *J* = 15.76, 8.51 Hz, 1H), 2.78 (dd, *J* = 15.76, 6.94 Hz, 1H), 2.87–2.98 (m, 2H), 3.24 (t, *J* = 6.62 Hz, 2H), 3.57 (dd, *J* = 8.51, 6.94 Hz, 1H); MS (EI) *m*/*z* 192, 115, 76]. This is a product of the addition reaction of the cysteamine thiyl radical to the double bond of fumaric acid, as shown in Figure S4, which is well known as the thiol–ene reaction. This is one of the representative reactions in click chemistry because it has high chemical selectivity and does not easily cause side reactions [18].

**FIGURE 3:**
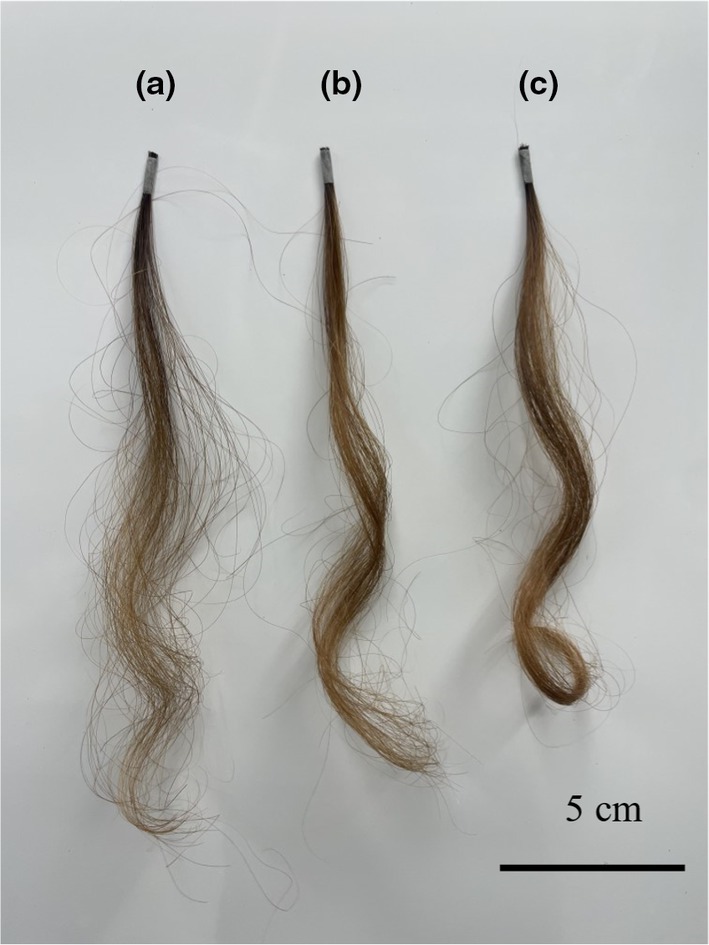
Results of curl retention test. (a) Treatment without ATS (blank), (b) treatment with 1.0% glyoxylic acid, (c) treatment with 1.0% ATS.

To confirm that ATS was the active ingredient, a curl retention test was performed using ATS solution. Hair samples (commercially available bleached hair) were treated with an aqueous solution of purified ATS, and the results were compared with those of samples treated with pure water without ATS (blank). After each treatment, samples exposed to 30°C and RH 90% environment for 1 h are shown in Figure 3. Figure 3a–c are blank (only water), 1.0% glyoxylic acid, and 1.0% ATS‐treated samples, respectively. The ATS‐treated sample retained its curl shape better than the blank and glyoxylic acid‐treated samples. The results of the curl retention measurements are presented in Table 1. Curl retention increased compared with that of the blank and glyoxylic acid treatments. These results indicate that ATS has an excellent hair‐styling effect and is effective in maintaining the shape during processing. We concluded that the hair‐straightening effect shown in Figure 1 was due to the action of ATS.

**TABLE 1:** Curl retention results.

	*L*/cm	*L*_0_/cm	*L*_t_/cm	Retention (%)
Blank	25.0	5.5	19.0	30.8
Glyoxylic acid	25.0	5.2	16.0	45.5
ATS	25.0	5.7	15.0	51.8

### Changes in hair shape due to ATS treatment

**FIGURE 4:**
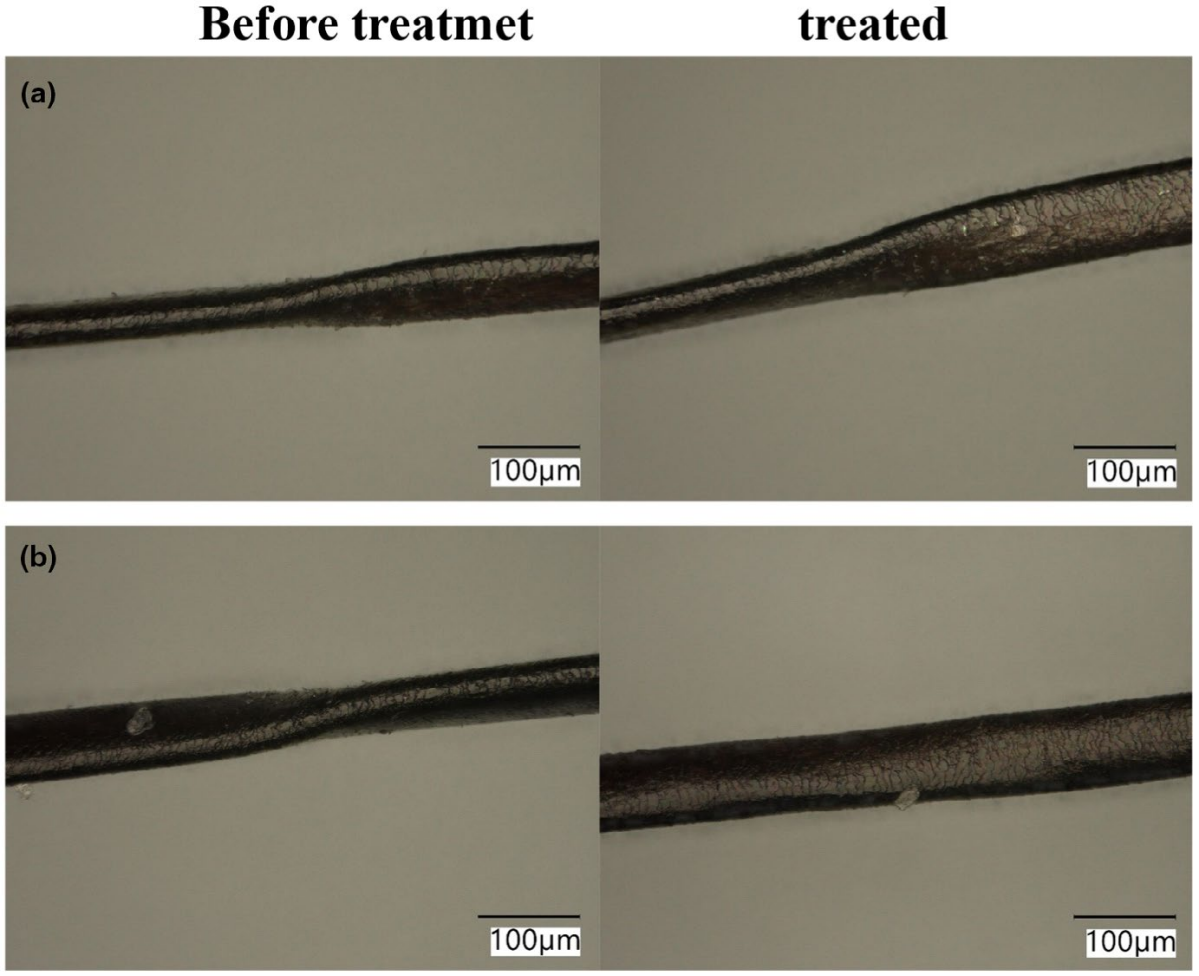
Microscopic observation of changes in flat spots of African hair due to ATS treatment: (a) without ATS (blank), (b) 0.5% ATS.

Figure 4 shows the changes in the shape of African hair before and after the ATS treatment under a microscope. Strongly curly hair has flat spots in places that may cause frizziness. The flat spots had been marked in advance on the glass plate, and changes in shape due to the treatment with pure water (without ATS, a) and treatment with 0.5% ATS solution (b) were compared. After the treatment, these were allowed to stand for 1 h in a constant temperature of 40°C and an RH of 90%. The flat spot did not change substantially during treatment without ATS, but the flat spot swelled and became nearly circular after ATS treatment. It is thought that ATS treatment eliminated flat areas of hair, making it appear straighter.

### Friction measurements

**FIGURE 5:**
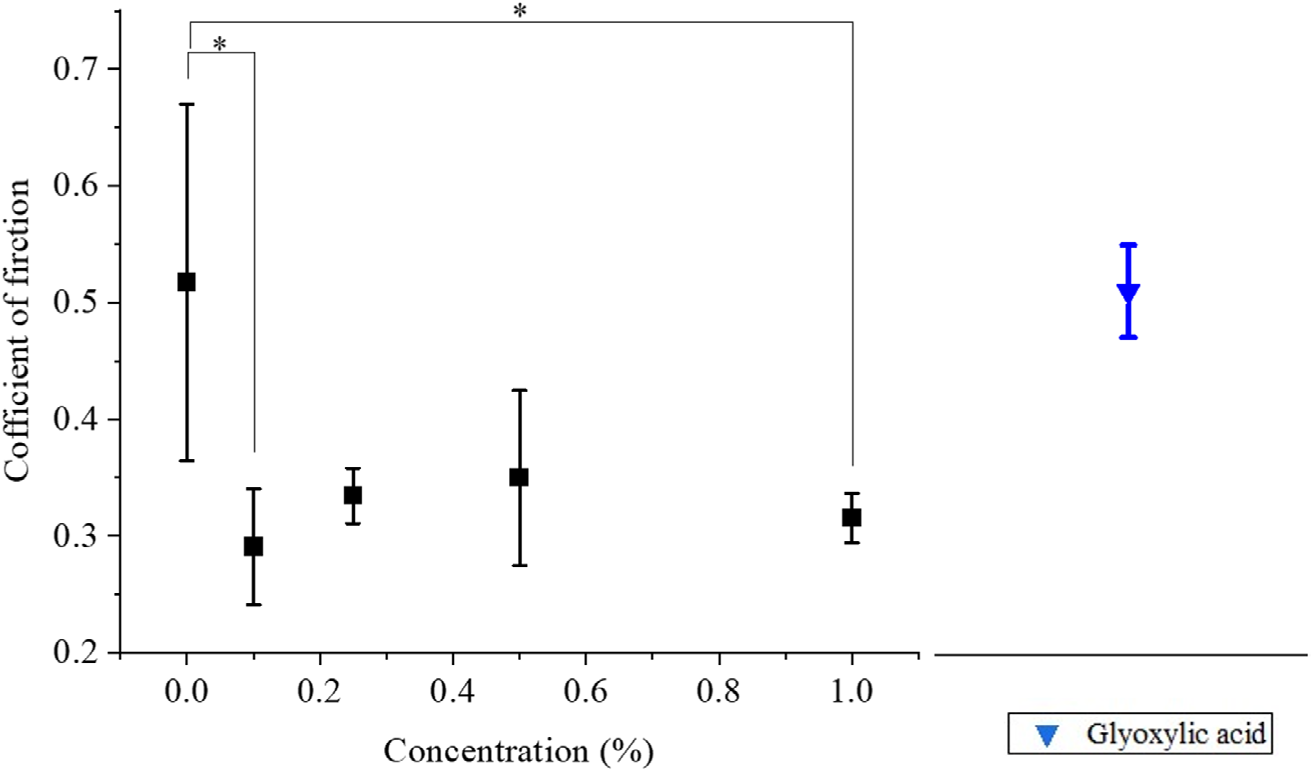
ATS concentration dependence of coefficient of friction. Treatment with glyoxylic acid was also performed for comparison. Bleached hair samples were treated by 0%–1.0% of ATS solution or other agents at 120°C hair iron. *p ≤ 0.05.

**FIGURE 6:**
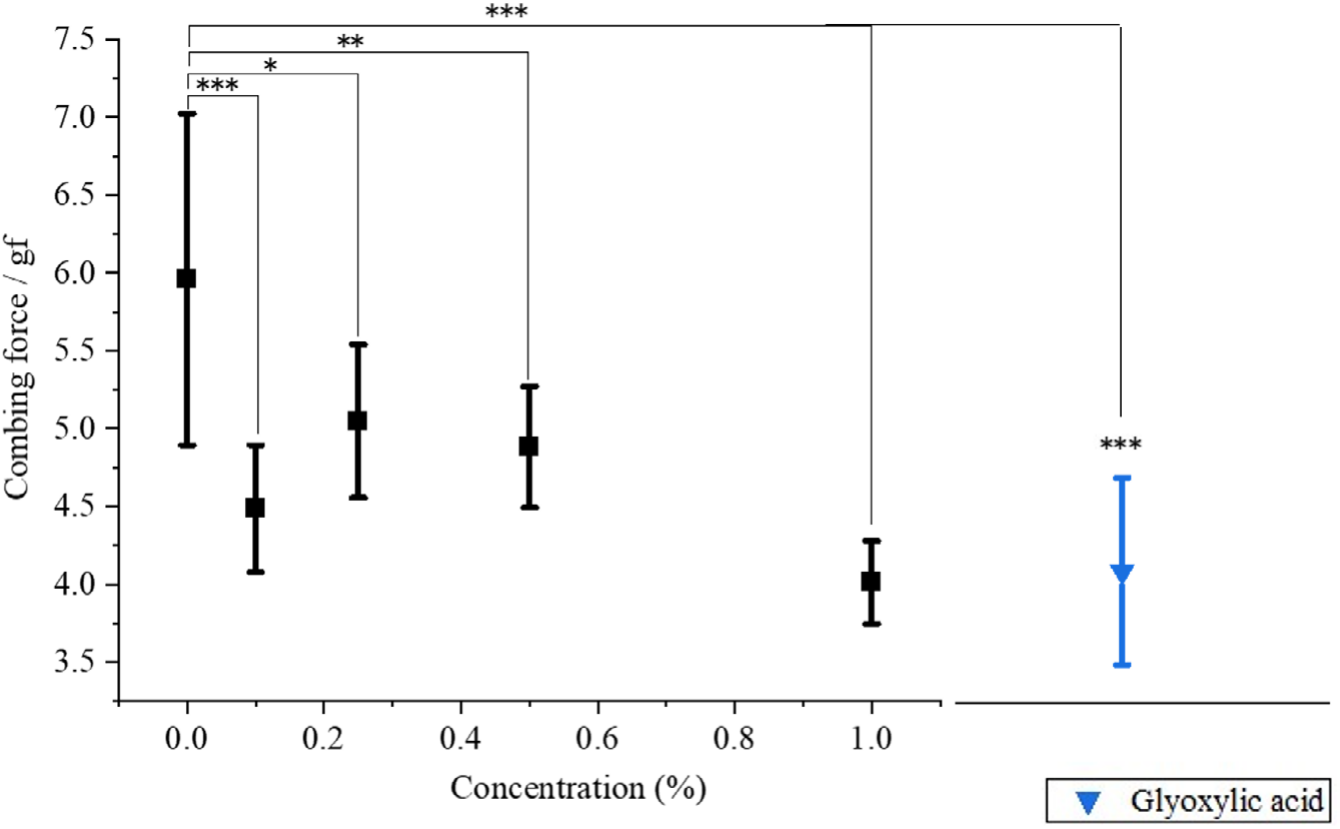
ATS concentration dependence of combing force. Treatment with glyoxylic acid was also performed for comparison. The samples were treated in the same manner as in the friction measurements. *p ≤ 0.05; **p ≤ 0.01; ***p ≤ 0.001.

The removal of flat spots, as described above, was expected to improve the feeling of touching the hair. Therefore, hair surface friction measurements were performed. Figure 5 shows the dependence of the friction coefficient on ATS concentration. The results are also shown for when glyoxylic acid was applied to hair alone. The results of the statistical analysis (performed using python and origin pro, OriginLab Corp. MA, USA) are also shown. Bleached hair was immersed in 0%–1.0% of ATS solution and heated to 120°C with a hair iron before being used for the measurements. While the average coefficient of friction of the blank (without ATS) was approximately 0.51, the sample treated with 0.1% ATS was approximately 0.3, and it did not change significantly even when the concentration was increased to 1.0%. Although no concentration dependence was observed, it is thought that 0.1% treatment caused the hair to swell and change into a nearly circular shape, reducing the coefficient of friction sufficiently. Treatment with glyoxylic acid did not reduce the coefficient of friction. Additionally, a combing test was performed on ATS‐treated hair bundles. Figure 6 shows the dependence of combing force on ATS concentration. The combing force of hair bundles of the treatment without ATS was 5.96 ± 1.06 gf, but the value was significantly reduced by the ATS treatment. Similar to the surface friction results, the combing test also showed a significant change with 0.1% treatment. It is thought that the ATS treatment reduced the coefficient of friction, making hair easier to comb. Treatment with glyoxylic acid alone also reduced combing resistance, although this was highly variable. This suggests that the ease of coming is not determined solely by the coefficient of friction. Hair curvature is known to be the most significant factor that improves combability [19], and glyoxylic acid, which is used in hair straightening and has hair improvement properties [7], may be responsible for the improved combability.

### Bending stiffness measurement

Bending stiffness was measured to quantify the firmness and stiffness of hair, and it has been reported that bleaching or UV irradiation increases bending stiffness compared to that of healthy hair [20].

Generally, the following formula (Equation 1) applies to bending moment.

(1) $$M=E∙\frac{I}{\rho }.$$

**FIGURE 7:**
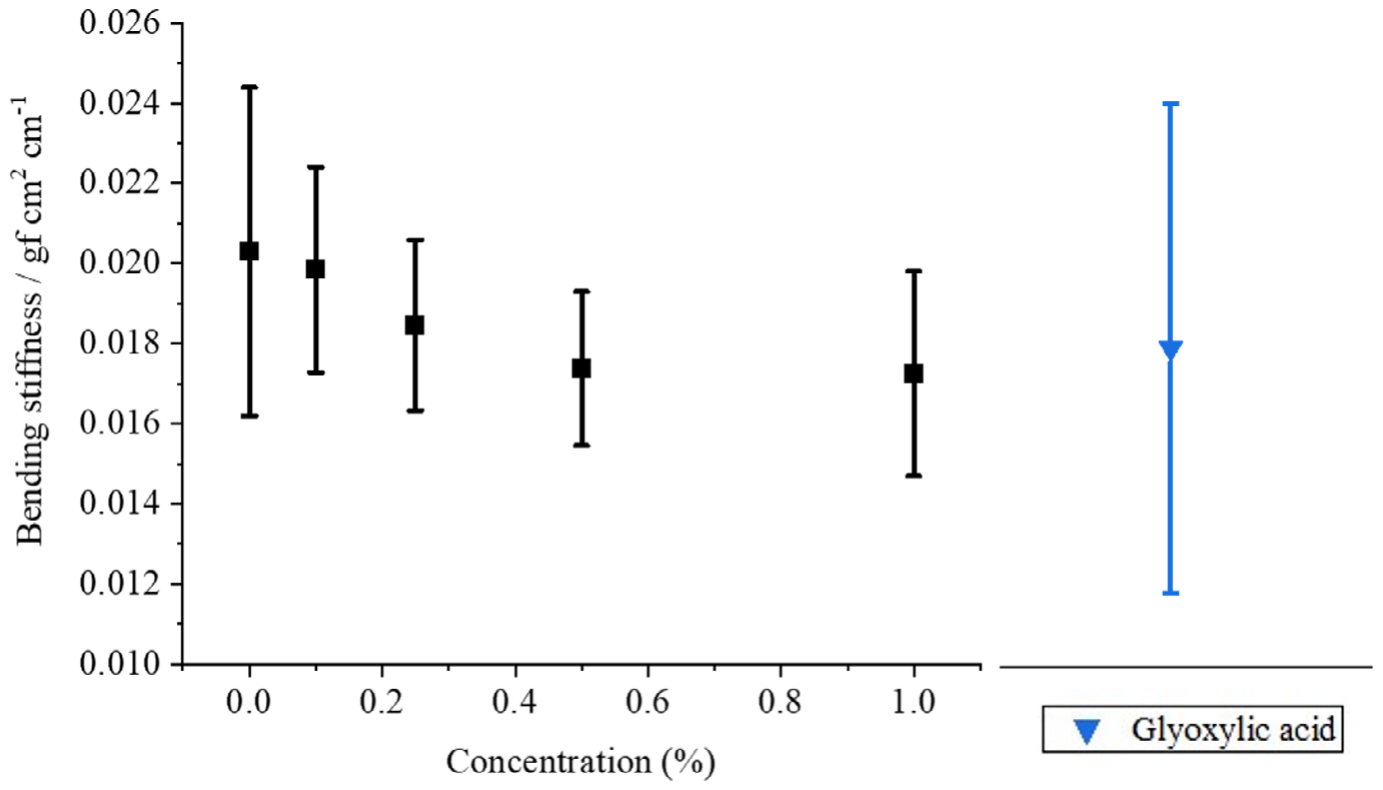
ATS concentration dependence of bending stiffness Treatment with glyoxylic acid was also performed for comparison. The samples were treated in the same manner as in the friction measurements.

Here, *M* is the bending moment, *E* is Young's modulus, *I* is the moment of inertia, and *ρ* is the radius of curvature. *E·I* is called bending rigidity, and the greater the bending rigidity, the smaller the curvature and the harder it is to bend. Figure 7 shows the ATS concentration dependence of the bending stiffness of the hair. The samples were treated in the same manner as for the friction measurements and comb tests. The value of blank test (without ATS) was 0.020 ± 0.0049 gf cm^2^ cm^−1^, whereas the value decreased with increasing ATS concentration, reaching 0.017 ± 0.0025 gf cm^2^ cm^−1^ when treated with 1.0% ATS. Although no statistical differences were found due to the large variation, a tendency for bending stiffness to decrease with ATS concentration was observed, suggesting that ATS has the effect of softening hair. However, no change in the hysteresis values was observed with respect to the ATS concentration. From these results, it can be concluded that hair treated with ATS becomes flexible and elastic. When the treatment with glyoxylic acid was performed, a tendency for the combing resistance to decrease was also observed, but there was a large variability, indicating instability of the treatment.

It is known that in bleached hair, the S–S bonds inside the hair are broken and the number of bonds decreases; however, this is not thought to be directly related to an increase in bending stiffness [20, 21, 22]. It is believed that the bleaching process causes the proteins in the matrix to denature and flow out, reducing the hygroscopicity within the hair and the hydrogen bonding between proteins, causing the hair to lose its flexibility [21]. Thus, these results suggest that ATS treatment increases the water content of hair.

### Moisture content measurement

The moisture content was measured by thermal analysis. In accordance with previous research [13], the weight loss up to 65°C was considered free water, and the weight loss at 180°C was considered bound water, and the sum of these was taken as the water content inside the hair. In this study, bleached hair was used, and a comparison was made between treatments without and with 1.0% ATS. The results of without ATS treatment (only pure water) were 10.31% ± 0.58% for free water and 1.83% ± 0.10% for bound water, and thus 12.15% ± 0.51% for total water content, whereas the sample treated with 1.0% ATS retained 10.80% ± 1.44% of free water, 1.89% ± 0.32% of the bound water, and total moisture content was 12.70% ± 1.6%. Although there was no statistically significant difference in the results of this measurement, there was a tendency for the water content to increase slightly in both free water and bound water. In general, bleaching processes oxidize hair components, such as melanin, and damage the chemical and physical structures of hair. These chemical treatments reduce the moisture content of hair, which can damage the hair fibre. Treatment with ATS is expected to reduce this loss of moisture and, therefore, bring about changes in the condition of the hair fibres. Another possible reason for the straightening of African human hair shown in Figure 4 is the swelling of the hair due to increased water content.

### Small‐angle X‐ray scattering (SAXS) measurement

**FIGURE 8:**
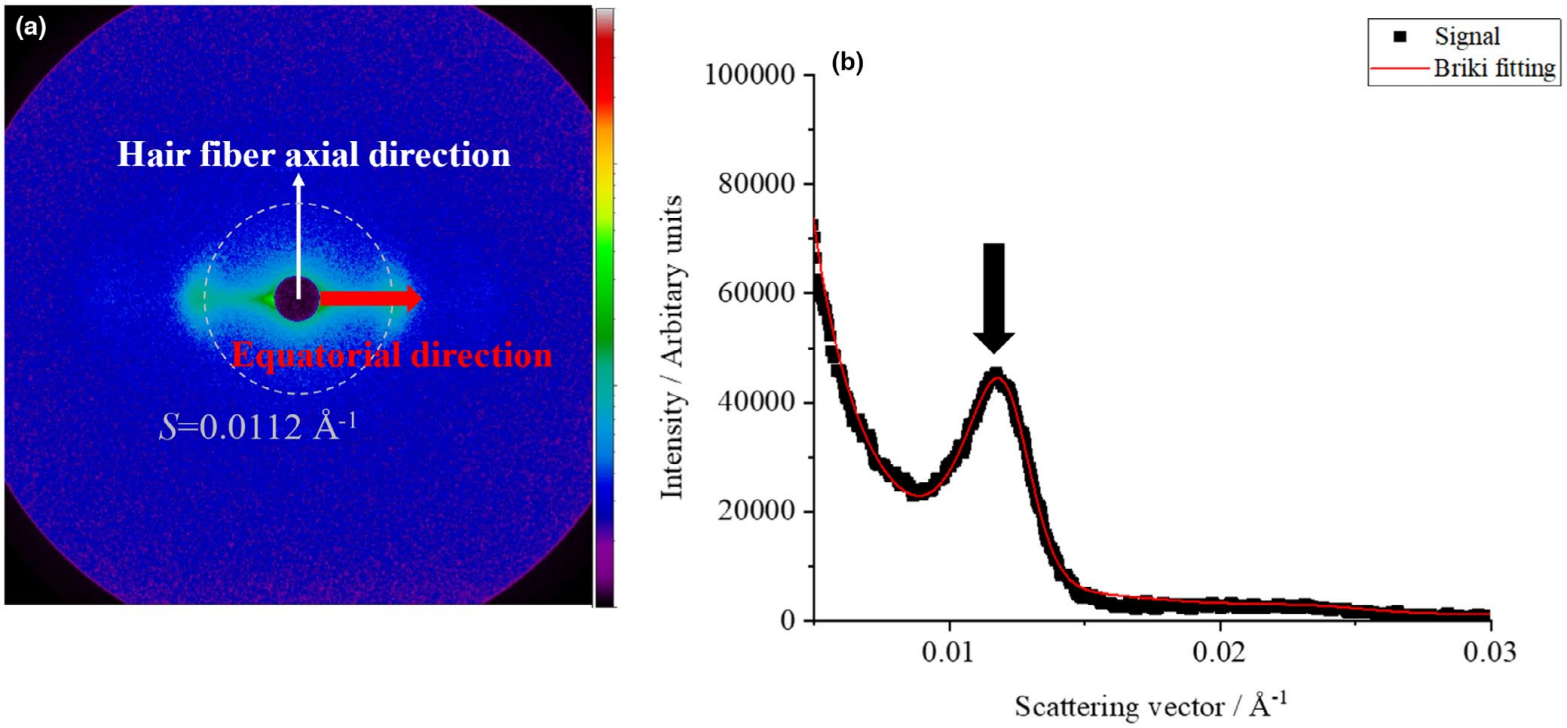
(a) 2D SAXS image from the cortex, (b) equatorial intensity profiles of bleached hair treated without ATS at room temperature.

The state of the IF was analysed using SAXS. Bleached hair treated with a hair iron at room temperature and at 60, 80, and 120°C with ATS concentrations of 0% to 1.0% was used for the measurements. SAXS images derived from the cuticle, cortex, and medulla layers were obtained from the outside of the hair. However, the characteristic SAXS image derived from the IF of the cortex layer was analysed in this study. A typical example of a 2D SAXS image obtained without ATS at room temperature is shown in Figure 8a. The direction of the hair axis is indicated by white arrows in Figure 8. The intensities obtained along the equatorial direction from the centre indicated by red arrow (90° ± 5°) are plotted as black circles in Figure 8b. We defined the scattering intensity as the scattering vector *S* (=2 sin *θ*/*λ*, where 2*θ* is the scattering angle, and *λ* is the X‐ray wavelength). From this profile, the IF radius *d* and the inter‐IF distance <*d*> were determined by performing least‐squares fitting using the Equation of Briki [15]. The scattering intensity *I*(*S*) is written in the following form:

(2) $$I\left(S\right)\propto {\left\{F\left(S\right)\right\}}^{2}Z\left(S\right)+\alpha e^{-\mathrm{\beta s}}.$$

Here, *F*(*S*) is a shape factor determined by the shape of IF, *Z*(*S*) is an interference function determined by the arrangement of IF, and $\alpha e^{-\mathrm{\beta s}}$ background scattering from sources other than IF. Assuming that IF is a homogeneous infinite cylinder and that the adjacent IF follows an existence probability that, in turn, follows a normal distribution in a hexagonal grid, each can be described by the following formulas:

(3) $$F\left(S\right)=2\pi \rho r^{2}J_{1}\left(2\mathrm{\pi rS}\right)/\left(2\mathrm{\pi rS}\right)$$

(4) $$Z\left(S\right)=\int z\left(x\right)e^{-2\text{piSx}}\;dx$$

Also, *z*(*x*) can be written as follows:

(5) $$z\left(x\right)=\delta \left(0\right)+\sum {\left\{G_{<d>,\sigma }\left(x\right)\right\}}^{*n}+\sum {\left\{G_{<d>,\sigma }\left(-x\right)\right\}}^{*n}$$

Here, *n* is *n* convolutions, and *δ* is a *δ* function *G*, which is a Gaussian function with a maximum value at <*d>* and a standard deviation *σ*. The experimental results were well fitted using Equation (2), as indicated by the red line in Figure 8b. From the fitting, *d* value and <*d*> were obtained.

**FIGURE 9:**
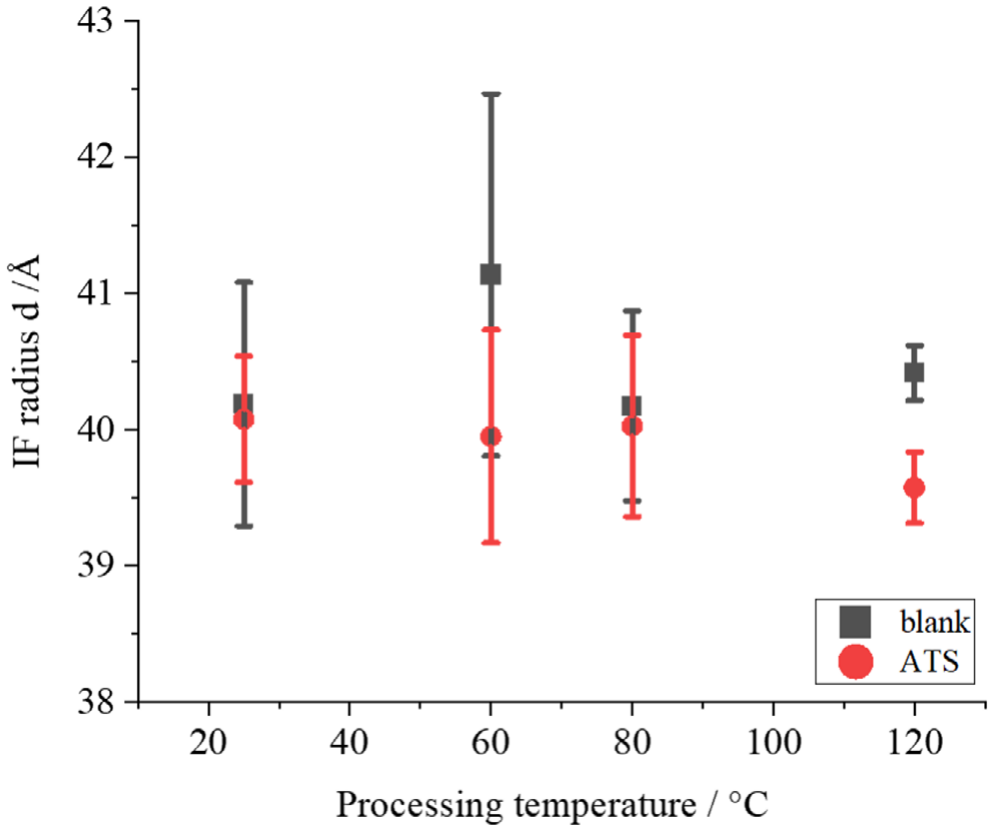
Treatment temperature dependence of IF radius, *d* of hair treated at 120°C.

**FIGURE 10:**
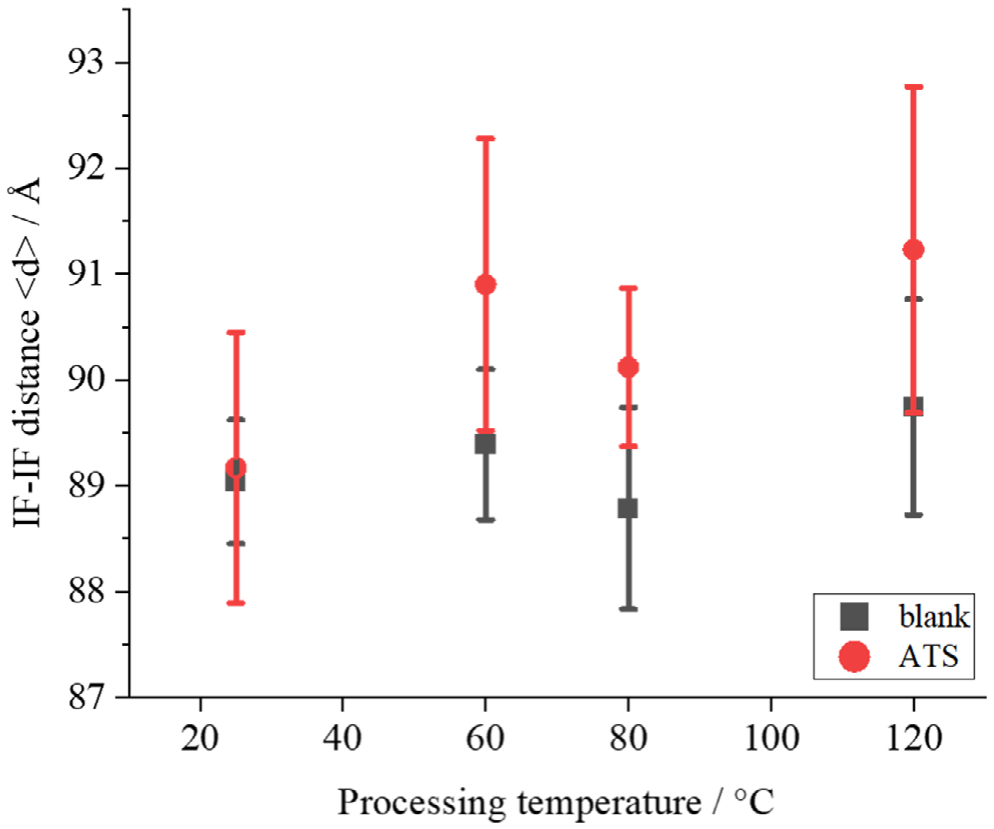
Treatment temperature dependence of IF–IF distances <*d*> for blank and ATS‐treated hair.

**FIGURE 11:**
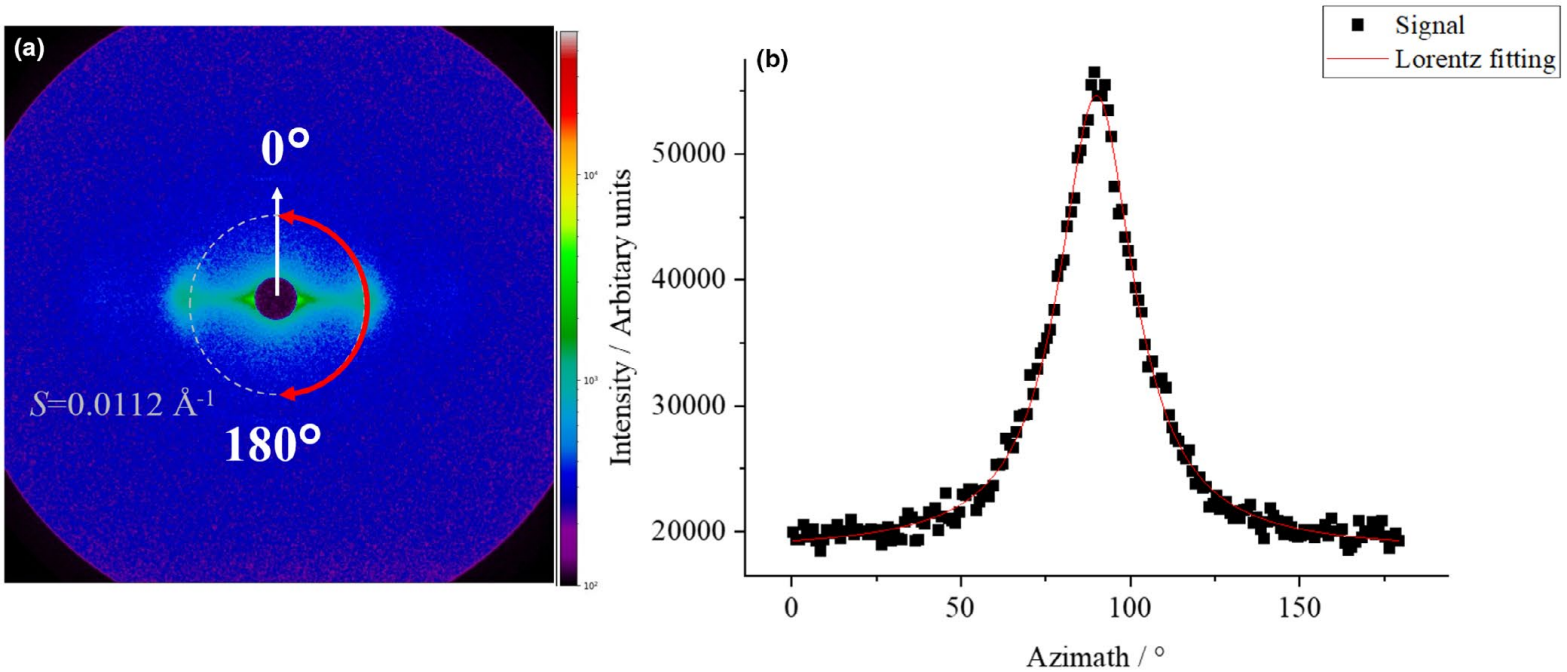
(a) 2D SAXS image from the cortex, (b) azimuthal scattering intensity profile of bleached hair treated without ATS at room temperature.

**FIGURE 12:**
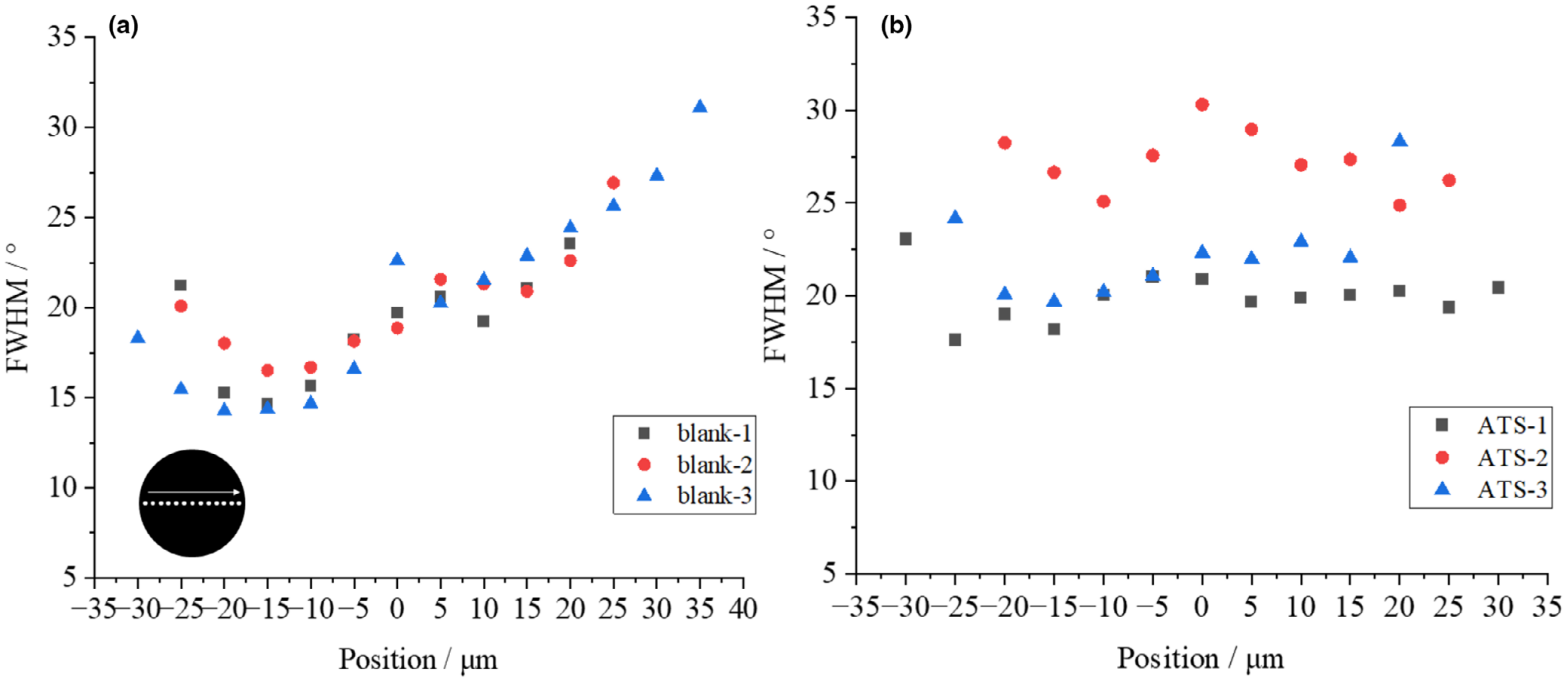
Variation in the thickness direction of average tilt angles of IF for (a) blank and (b) 1.0% ATS treated at 120°C.

Figure 9 shows the treatment temperature dependence of *d* for 1.0% ATS concentration. Although no significant difference was obtained owing to large variations, *d* tended to decrease as the ATS concentration increased. This result suggests that ATS affects IF, although there is a large degree of variability due to individual differences in hair. Figure 10 shows the treatment temperature dependence of *<d>* without ATS addition (blank) and with 1.0% ATS. When the treatment without ATS was performed, no temperature dependence of <*d*> was observed. On the other hand, when 1.0% of ATS was added, as indicated by the red circle, a tendency for *<d>* to expand with increasing treatment temperature was observed, although no statistically significant difference was obtained. In addition, the average tilt angle of the IFs was determined from SAXS measurements. As shown in SAXS image in Figure 11a, the fibre axis of the sample was set as 0°, and the intensity profile at the azimuthal angle near the specific scattering angle (*S* = 0.0112 Å^−1^) was plotted up to 180°, as indicated by the red arrow. As indicated by the black arrow in Figure 8b, the signal at *S* = 0.0112 Å^−1^ was considered to be derived from the inter‐IF distance. The intensity profiles are shown in Figure 11b. The profile was well fitted with a Lorentzian function, and the average tilt angle of the IF with respect to the hair fibre axis was estimated from the value of its full‐width at half maximum (FWHM) [16, 17]. Figure 12 shows the change in the average IF tilt angle in the thickness direction of hair from SAXS images measured every 5 μm in the thickness direction. For both the blank treatment (without ATS, Figure 12a) and the ATS 1.0% treatment (Figure 12b), both of which were treated at 120°C, the cross sections of three hairs were measured and plotted. The horizontal axis represents the center position of the hair at 0. In the blank treatment, the FWHM values (i.e. the slope of IF) differed greatly depending on the position, whereas in the hair treated with ATS, the difference in the slope of IF depending on the measurement position was small. In other words, the non‐uniformity of the IF arrangement was corrected by treatment with ATS. Recent studies have reported that the arrangement of IF is different between curly hair and straight hair, and it has also been reported that reduction treatment with a perming agent and bleaching treatment also affect the orientation of IF [23, 24]. In hair straightening, heat treatment reduces the crystallinity of IF and causes it to swell in the direction perpendicular to the fibre axis. This swelling behaviour of the matrix is caused by the hair straightening treatment with glyoxylic acid [25] and changes in humidity [26]. A correlation exists between hair swelling and hair straightening [1]. This suggests that one of the reasons for the straightening of African human hair shown in Figure 4 is the widening of the distance between IFs and the improvement of the alignment. Boga et al. concluded that glyoxylate forms imine bonds inside hair via a reversible reaction between the carbonyl groups and nucleophilic sites on the side chains of keratin‐constituting amino acids [7, 27].

Although no difference was observed in the IF diameter due to ATS treatment, there was a tendency for the distance between the IFs to widen, suggesting swelling. It was also observed that the variation in the average tilt angle of the IFs was reduced, and it can be inferred that ATS has the effect of making the IFs parallel to each other and neatly arranged. These results suggest that ATS forms a cross‐linked structure within the matrix, which is believed to help maintain the hair shape.

## CONCLUSION

ATS, which is produced by the reaction of cysteamine and fumaric acid, is a highly effective shape‐control substance that can straighten extremely curly hair long‐lasting. In the bending test, a decrease in bending stiffness was observed, indicating an improvement in flexibility. The friction coefficient also decreased, indicating a change in the shape and a qualitative change in the surface condition of the hair. SAXS measurements showed that ATS treatment widened the distance between IFs inside the hair and improved their orientation. This treatment was thought to cause the matrix region, which is the interstitial substance of IF–IF, to swell, thereby increasing the hair texture. In conjunction with the results of the water content measurement, the increased IF distance increased the moisture absorption area of the matrix and weakened the hydrogen bonds between the molecules and proteins, resulting in a decrease in the friction coefficient and bending stiffness, which led to changes in the physical properties of the hair. A long‐lasting shape‐control effect of ATS was demonstrated, and various measurements revealed that it acted on the matrix region. In future, we plan to conduct more detailed molecular studies.

## CONFLICT OF INTEREST STATEMENT

The authors declare that there are no conflicts of interest regarding the publication of this paper.

## CONSENT

This manuscript has not been published or presented elsewhere in part or entirety and is not under consideration by another journal. All study participants provided informed consent.

## Supporting information


Data S1.
