# Supplementary material for: Synthesis of a new amino acid derivative with long‐lasting hair shape control effects and elucidation of its mechanisms
Source: Int J Cosmet Sci. 2025 Feb 18;47(4):585–96. doi: 10.1111/ics.13054 (PMC12319485; doi:10.1111/ics.13054)
Supplement: Supplementary file 1 — Data S1. [file ICS-47-585-s001.docx]

Supporting Information

**Synthesis of a New Amino Acid Derivative with Long-Lasting Hair Shape Control Effects and Elucidation of its Mechanisms.**

**Figure S1.** An example of bending moment-curvature curve of hair.


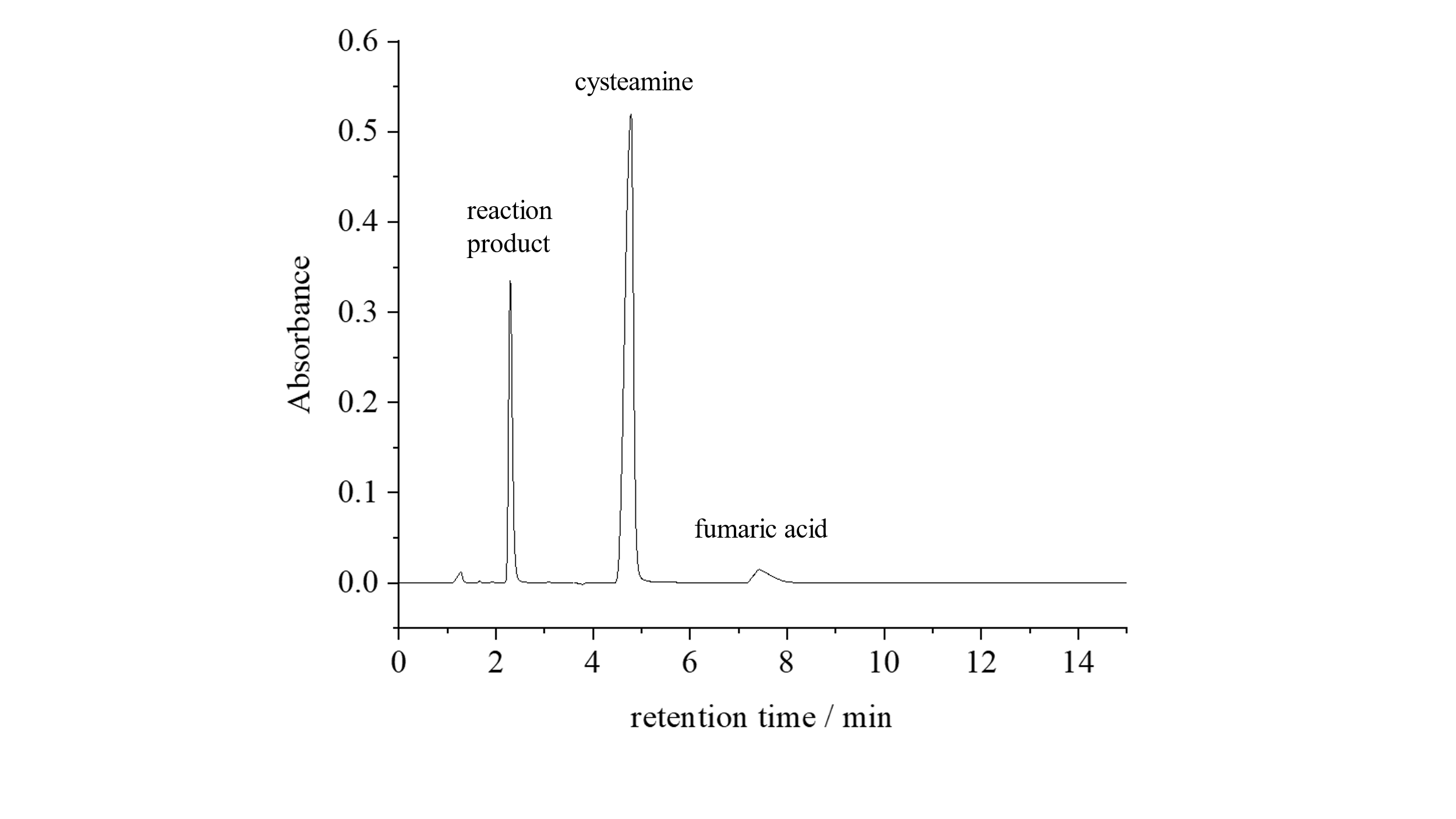


**Figure S2.** The chromatogram of the reaction solution of cysteamine and fumaric acid.


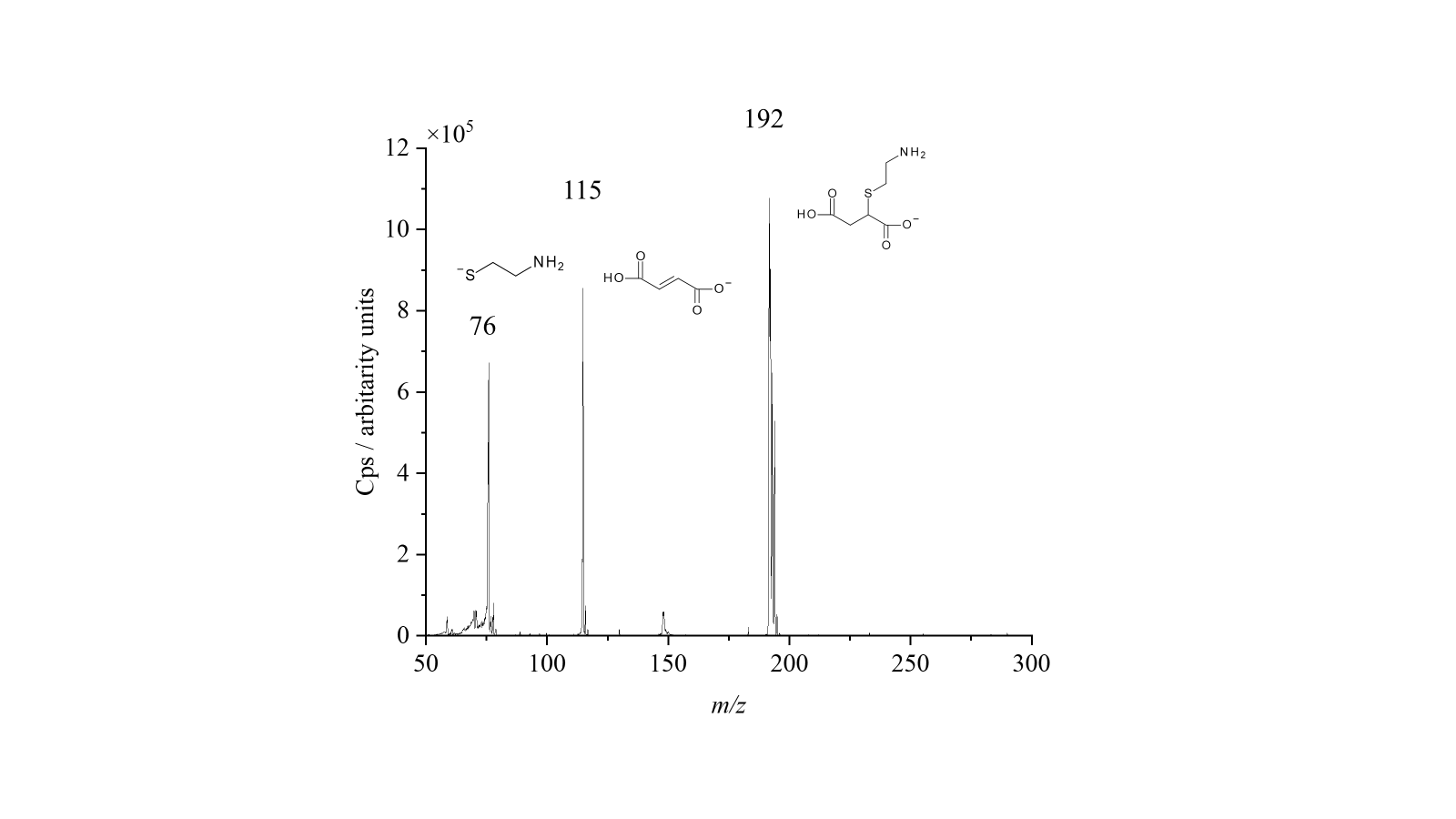


**Figure S3.** The ESI-quadrupole mass spectrum obtained at 2.2 min of the chromatogram of Fig. S2.


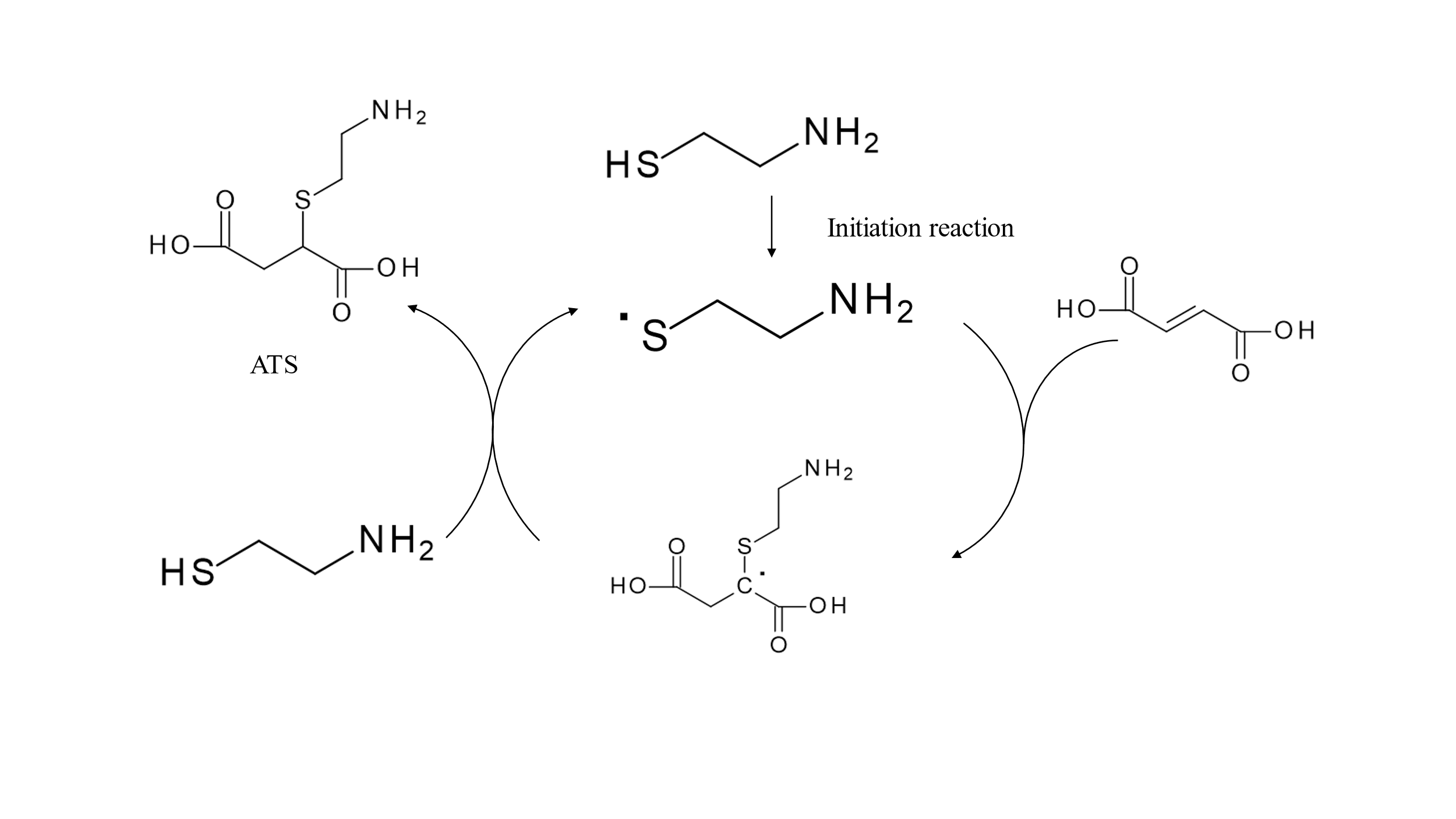
**Figure S4.** The proposed reaction mechanism of cysteamine and fumaric acid.

Cysteamine thiyl radical is added to fumaric acid double bond, the resultant fumaric acid-cysteamine adduct radical reduced by cysteamine to produce ATS and a cysteamine thiyl radical. The chain reaction will be terminated by the coupling reaction between cysteamine thiyl radicals.
